# Cervical Dystonia: A Disorder of the Midbrain Network for Covert Attentional Orienting

**DOI:** 10.3389/fneur.2014.00054

**Published:** 2014-04-28

**Authors:** Michael Hutchinson, Tadashi Isa, Anna Molloy, Okka Kimmich, Laura Williams, Fiona Molloy, Helena Moore, Daniel G. Healy, Tim Lynch, Cathal Walsh, John Butler, Richard B. Reilly, Richard Walsh, Sean O’Riordan

**Affiliations:** ^1^Department of Neurology, St. Vincent’s University Hospital, Dublin, Ireland; ^2^School of Medicine and Medical Science, University College Dublin, Dublin, Ireland; ^3^Department of Developmental Physiology, National Institute for Physiological Sciences, Okazaki, Japan; ^4^Department of Neurophysiology, Beaumont Hospital, Dublin, Ireland; ^5^Cork University Hospital, Cork, Ireland; ^6^Department of Neurology, Beaumont Hospital, Dublin, Ireland; ^7^Dublin Neurological Institute, Mater Misericordiae Hospital, Dublin, Ireland; ^8^Department of Statistics, Trinity College Dublin, Dublin, Ireland; ^9^Trinity Centre for Bioengineering, Trinity College Dublin, Dublin, Ireland; ^10^Department of Neurology, The Adelaide and Meath Hospital, Dublin, Ireland

**Keywords:** cervical dystonia, temporal discrimination, covert attention, GABA, superior colliculus

## Abstract

While the pathogenesis of cervical dystonia remains unknown, recent animal and clinical experimental studies have indicated its probable mechanisms. Abnormal temporal discrimination is a mediational endophenotype of cervical dystonia and informs new concepts of disease pathogenesis. Our hypothesis is that both abnormal temporal discrimination and cervical dystonia are due to a disorder of the midbrain network for covert attentional orienting caused by reduced gamma-aminobutyric acid (GABA) inhibition, resulting, in turn, from as yet undetermined, genetic mutations. Such disinhibition is (a) *subclinically* manifested by abnormal temporal discrimination due to prolonged duration firing of the visual sensory neurons in the superficial laminae of the superior colliculus and (b) *clinically* manifested by cervical dystonia due to disinhibited burst activity of the cephalomotor neurons of the intermediate and deep laminae of the superior colliculus. Abnormal temporal discrimination in unaffected first-degree relatives of patients with cervical dystonia represents a subclinical manifestation of defective GABA activity both within the superior colliculus and from the substantia nigra pars reticulata. A number of experiments are required to prove or disprove this hypothesis.

## Introduction

Cervical dystonia, characterized by sustained or intermittent neck muscle contractions causing abnormal head movements, is the most common form of adult-onset idiopathic isolated focal dystonia (AOIFD) and may be sporadic or familial (1). The pathogenesis of cervical dystonia is unknown (2). In patients with cervical dystonia, a number of neurophysiological and neuroimaging abnormalities have been described as endophenotypes implying a relationship to causal mechanisms [reviewed in Ref. (3)]. We have argued that many of these abnormalities are secondary endophenotypes resulting from disease manifestation; only mediational endophenotypes, including abnormal temporal discrimination, found in unaffected first-degree relatives as well as patients, are relevant to understanding disease pathogenesis (4, 5) (Table 1). The features of a mediational endophenotypes are (a) it is an expression of a genetic mutation, necessarily present prior to disease onset, (b) it reflects disease susceptibility and is not altered by disease expression or severity, and (c) it is more penetrant than the phenotype (6). The attributes of abnormal temporal discrimination which indicate that it is a mediational endophenotype of cervical dystonia are listed in Table 1.

**Table 1:** **A summary of the evidence from experimental clinical and laboratory studies suggesting that an abnormal TDT is a mediational endophenotype of cervical dystonia**.

**Sensitivity:** the TDT is abnormal in more than 80–90% of patients with various idiopathic AOIFD phenotypes, and sensitivity is 97% in cervical dystonia (7)
**Specificity:** the specificity of an abnormal TDT is 98–100% (5, 7)
**Autosomal dominant transmission:** abnormal TDTs show autosomal dominant transmission in multiplex AOIFD families (8) and in families of sporadic cervical dystonia (9). Unaffected obligate heterozygotes have abnormal TDTs (8)
**Age-related and gender-related penetrance:** in unaffected first-degree female relatives, an abnormal TDT is fully penetrant by 48 years of age. In male relatives, there is 40% penetrance after 25 years of age (5)
**Putaminal enlargement:** unaffected relatives (of patients with cervical dystonia) with abnormal TDTs have larger putaminal volumes by VBM than relatives with normal TDTs (8). Putaminal enlargement is found in patients with blepharospasm (10) and musicians with task-related dystonia (11)
**Putaminal activation:** unaffected first-degree relatives (of patients with cervical dystonia) with abnormal TDTs have reduced putaminal activation in a temporal discrimination test compared to relatives with normal TDTs (5). Perceptual certainty in a temporal discrimination task is associated with putaminal activation; the putamen is involved early in a temporal processing task (12)

*AOIFD, adult-onset isolated focal dystonia; TDT, temporal discrimination threshold; VBM, voxel-based morphometry*.

Mediational endophenotypes may elucidate mechanisms of disease pathogenesis not obvious from the phenotype. Shared pathological mechanisms for both disordered temporal discrimination and cervical dystonia have become evident over the past 10 years from highly sophisticated piecemeal investigations in diverse disciplines including primate and sub-primate neurophysiology, anatomy, chemistry, psychology, and clinical research. Using this evidence, we propose a unified theoretical model to explain the etiopathogenesis of cervical dystonia.

## Hypothesis

We postulate that cervical dystonia is a disorder of a midbrain network for covert attentional orienting involving both the sensory and motor laminae of the superior colliculus. The mediational endophenotype, abnormal temporal discrimination and the phenotype, cervical dystonia, are caused by defective inhibition of sensory and motor neurons in the superior colliculus. This disinhibition is due to the effects of reduced gamma-aminobutyric acid (GABA) activity, both from the substantia nigra pars reticulata (SNpr) and GABAergic interneurons within the superior colliculus. Abnormal temporal discrimination, a subclinical marker of this disinhibition, results from prolonged duration firing of visual sensory neurons in the superficial layers of the superior colliculus (SLSC). The abnormal involuntary movement characteristic of cervical dystonia results from *subsequent secondary* disinhibition of cephalomotor neurons in the intermediate and deep layers of the superior colliculus (DLSC). A summary of the arguments deployed in support of this hypothesis is outlined in Table 2.

**Table 2:** **An outline summary of some of the more important experimental evidence from clinical and animal studies supporting the hypothesis that both abnormal temporal discrimination and adult-onset isolated focal dystonia are due to a disorder of reflex covert orienting caused by defective GABAergic inhibition in the superior colliculus**.

**Hypothesis:** pathological disinhibition in the midbrain network for covert attentional orienting due to deficient GABAergic activity causes
(a) *Subclinically*, abnormal temporal discrimination due to disordered visual processing in the SLSC
(b) *Clinically*, cervical dystonia due to disinhibited prolonged burst activity of cephalomotor neurons in the DLSC
**Key observations in support of the hypothesis**
***Sensory aspects***
(1) The midbrain network for covert attentional orienting is a primitive, highly conserved system for detecting salient environmental change, inspecting the change, and responding rapidly and appropriately
(2) Wide field visual sensory neurons in the SLSC, via the retinotectal pathway, detect environmental visual change and respond by transient discharges to the pre-motor neurons for saccade generation and head turning
(3) Focal inactivation of the SLSC causes loss of covert visual attention in the visual field represented by the inactivated part of the SLSC
(4) Inhibitory GABAergic activity (within the superior colliculus and from the SNpr) limits the duration of the transient response in visual sensory neurons in the SLSC
(5) A moving or sudden luminant visual stimulus elicits a time-locked electromyographic response in cervical muscles involved in ipsilateral head turning
***Motor aspects***
(1) Movement is initiated by the striatum through release from the tonic inhibition exerted by the SNpr. A core neurophysiological feature of dystonia is reduced inhibition at all levels of the CNS; the most probable cause is defective GABAergic inhibition
(2) The oculomotor and cephalomotor pre-motor neurons of the DLSC for saccade generation and head turning are tonically inhibited by the SNpr. Release from that inhibition allows prolonged burst discharges of premotor neurons
(3) Oculomotor premotor neurons are gated by omnipause neurons; the cephalomotor premotor neurons are not gated
(4) Stimulation of cephalomotor neurons in the DLSC causes ipsilateral head turning in monkeys via the tectospinal and tecto-reticulospinal fiber tracts terminating in the cervical cord
(5) A unilateral lesion of the SNpr in macaques causes a movement disorder resembling cervical dystonia. A further lesion in the superior colliculus abolishes/attenuates the movement disorder

*This argument is expanded and fully referenced in the text. AOIFD, adult-onset isolated focal dystonia; DLSC, intermediate and deep laminae of the superior colliculus; SLSC, superficial laminae of the superior colliculus; SNpr, substantia nigra pars reticulata; TDT, temporal discrimination threshold*.

## Midbrain Network for Covert Attentional Orienting

Attentional networks in the brain can be divided into: (i) an overt (top-down) attentional network utilizing volitional eye movements to orient the visual system and (ii) a covert (bottom-up) network, which shifts attention to locations by head movements with or without saccade initiation (13). The midbrain covert attentional network captures change in the environment and alerts the individual to a salient stimulus, which requires inspection and action that may be important for survival. A significant node in this network is the superior colliculus, a complex sensorimotor brainstem structure, which acts as a sentinel for detecting sudden environmental change and responding to that change. The superior colliculus integrates multimodal sensory information from visual, auditory, and tactile sources; generates outputs for gaze, head, and arm movement; and sends priority signals to the substantia nigra pars compacta and the intralaminar nucleus of the thalamus (14).

## Superior Colliculus – Anatomy

The superior colliculus is a laminated structure with seven alternating gray and white layers (15). The SLSC, laminae I–III, receive direct visual input from the retina and indirect visual input from many cortical structures. The DLSC, laminae IV–VII, contain sensory cells responsive to multiple sources and premotor neurons with outputs to a number of structures described below. A significant intrinsic population of GABAergic inter-neurons (16) modulates the activity of both the visual sensory receptive cells in the SLSC and the premotor neurons in the DLSC (17, 18).

## Superior Colliculus – Inputs and Outputs

### Sensory inputs

The SLSC contain sensory cells, which receive visual inputs from the visual cortex and the retinotectal pathway. Magnocellular retinal ganglion cells convey responses to luminance change and movement in the visual field via the retinotectal pathway; about 10% of retinal ganglion cells project to the SLSC (19). It has been shown that the visual sensory cells of the SLSC also respond to chromatic S-cone stimuli, but at a longer latency than the faster retinotectal input; this longer latency reflects the retino-geniculo-cortical–collicular pathways (20–22). Among a variety of sensory neurons in the SLSC, wide field sensory neurons respond to salient environmental luminance change and movement; most neurons in the SLSC show an early 40–70 ms transient response to visual stimuli (23). Wide field sensory neurons in the SLSC have direct interlaminar connections with the premotor neurons in the DLSC considered to be the direct visuomotor pathway responsible for orienting responses (saccade generation and head turning) at extremely short latency (discussed below) (24, 25).

The SLSC is retinotopically organized; focal inactivation of a visual sensory area in the SLSC prevents macaques from detecting an odd-ball salient stimulus in the inactivated visual field and biases saccade selection away from that area (26, 27). Conversely, electrical stimulation of a focal area of the SLSC improves performance in a visual attention task in monkeys and favors saccades directed to the visual field corresponding to the stimulation site, indicating evidence for a role of the superior colliculus in the control of attention (28–30).

Although most experimental work relates to the visual system, deeper laminae of the superior colliculus receive other salient tactile and auditory inputs (15). Thus, both the SLSC and the DLSC receive multisensory inputs relating to environmental change, detect that change, and integrate the sensory information into a required appropriate motor output for immediate inspection or avoidance through motor outputs to the eye, neck, and arm muscles.

### Cholinergic inputs to the superior colliculus

Cholinergic inputs from the pedunculopontine tegmental nucleus to the intermediate layers of the superior colliculus primarily induce excitation of the motor output (31). When nicotine was applied *in vitro* to a rodent superior colliculus, with stimulation from the optic tract, sub-threshold excitatory post-synaptic potentials, recorded in the DLSC neurons, were depolarized and exhibited bursting responses (25). Nicotine injected into the superior colliculus of macaques had been shown to increase the frequency of express saccades with a shortened saccadic reaction time of approximately 100 ms (32). Recently, it has been demonstrated that the major function of the cholinergic input to the GABAergic neurons in the DLSC is excitatory and this may regulate the spike timing of the oculomotor and cephalomotor premotor neurons (33).

### Substantia nigra pars reticularis input to the superior colliculus

Since mid-1980s, it was indicated that the striato-nigro-collicular pathway is one of the neural circuits through which the basal ganglia can influence both ocular and cephalic motricity. The SNpr provides a tonically active GABAergic inhibitory outflow from the striatum to the DLSC (34, 35). In rats, stimulation of the SNpr causes both short latency and short duration inhibition in the response of the tectospinal neurons to spontaneous and peripherally evoked discharges (36). Tectospinal neurons discharge vigorously in response to SNpr silencing by intranigral GABA. Intrastriatal injection of glutamate, which silences SNpr, causes the tectospinal cells to discharge. This striatally induced firing of the tectospinal tract is sensitive to a GABA antagonist, intranigral bicuculline (37). In monkeys, a substantial number of neurons in the medial SNpr are concerned with orienting behavior including postural and motor mechanisms during visually triggered arm movements, (38) whereas the lateral part is involved with oculomotor control (34).

## Motor Output of Superior Colliculus

While the classical concept of the motor function of the superior colliculus is that of a saccade generator, recent studies in macaques have highlighted other motor functions.

### Saccade generation (oculomotor premotor neurons)

Saccades are driven by brief, high-frequency, bursts from the DLSC to the brain stem burst generator (39). This oculomotor drive is potently inhibited by the pontine omnipause neurons. These tonically prevent the premature execution of eye movements until the DLSC output reaches a threshold, which is usually associated with the high-frequency burst. Low-frequency DLSC activity does not influence eye movement because of this gating by the omnipause neurons (40). Visually guided saccades have latencies of 150–350 ms; both humans and monkeys can generate express saccades with latencies of 70–100 ms in monkeys and 80–120 ms in humans (41).

### Head-turn generation (cephalomotor premotor neurons)

**Figure 1:**
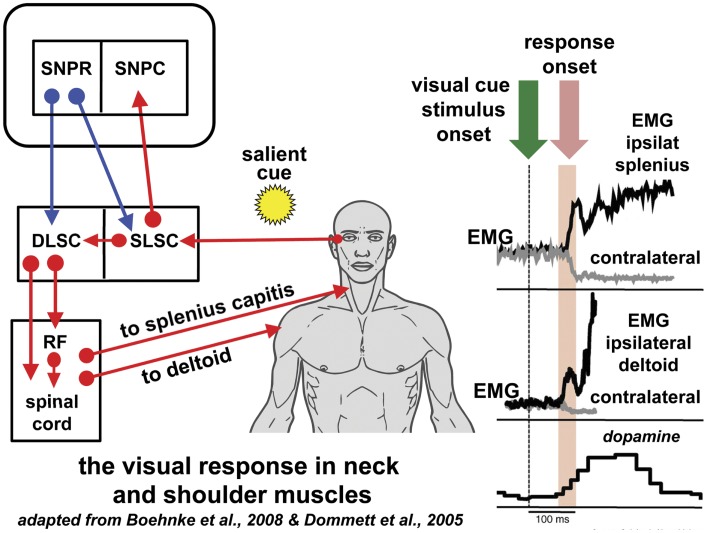
**Reflexive covert orienting: the visual grasp reflex**. This figure illustrates the postulated pathway for the visual grasp reflex in man. In a macaque, time-locked EMG responses recorded in the ipsilateral splenius capitis and deltoid, occur 80–90 ms following the sudden appearance of a cue in the visual field in a test of reflexive covert orienting (44). Responses are most marked when there is a gap of 200 ms between the fixation target and onset of the cue. Note that the EMG responses in the contralateral muscles diminish at the same time as the ipsilateral muscle activity increases. The onset of a saccade is not invariable and, if it does occur, the neck and shoulder muscle activity may precede saccade onset. The short latency phasic dopamine response is also included indicating the effect of salient events signaled to the substantia nigra pars compacta [adapted from Ref. (56)]. The postulated basal ganglia and brainstem pathway for this time-locked response is discussed in the text. Blue arrows indicated inhibition; red arrows indicate excitation. Figure adapted from Ref. (44) with permission. DLSC, intermediate and deep laminae of the superior colliculus; EMG, electromyographic; RF, reticular formation; ipsilat, ipsilateral; contralat, contralateral; SLSC, superficial laminae of superior colliculus; SNPR, substantia nigra pars reticulata; SNPC, substantia nigra pars compacta.

Initial studies in cats demonstrated that stimulation of the DLSC elicited early head movements without saccades (42) (Figure 1). In macaques, a number of experiments in the last 15 years have revealed that the DLSC contains premotor neurons solely concerned with head-turn generation; this motor pathway has not been examined in human. In macaques, the sudden presentation of a bright target in the temporal visual field stimulates a discharge of DLSC premotor neurons and a time-locked burst of activity in ipsilateral neck muscles with an extremely short latency (55–95 ms after the visual stimulus), regardless of the ensuing saccadic reaction time (43, 44) (Figure 1). This has been termed the “*visual grasp reflex*” (44). When a gaze shift (head plus eye movement) does follow, the neck muscle electromyographic response increases significantly prior to the higher velocity head movement associated with the gaze shift reflecting a correlation with movement preparation (45, 46). Low-frequency stimulation of DLSC neurons generates head turns without saccades in macaques and, in one study, 26% of DLSC neurons sampled were head-only neurons (42, 45, 47–50). In contradistinction to the oculomotor pathway, an absent or significantly weaker gating mechanism on the head pathway permits the generation of head-only movements that can precede gaze shifts (48, 51–53). Monkeys trained to make head-only movements to reward (without gaze shifts) showed DLSC neuronal bursts 20 ms before neck EMG activity (46); the classical DLSC neurons involved in gaze shift (head and eye movement) are not active in such head-only movements (54). In the sub-human primate, the representation of the position of head on body in the superior colliculus is used to compute oculomotor movement in saccade execution (55).

### Upper limb motor control from the superior colliculus

The primate superior colliculus also contains neurons which, when stimulated, produce arm movements independent of gaze shift (57–59); these neurons affect the shoulder and proximal arm muscles (57) and even hand muscles (60). In humans performing a reaching task with one arm following the sudden appearance of a visual target stimulus, there was a time-locked response in shoulder muscles at a latency of <100 ms indicating a rapid neural pathway linking visual input to arm motor out; this seemed most effective during reflexive movement tasks (61). The DLSC is the primary source of this short latency response.

### Other superior colliculus output targets

These targets include substantia nigra pars compacta, SNpr, and the intralaminar nucleus of the thalamus (14, 62).

## Anatomy of the Pathways for the Head-Turn Response to Superior Colliculus Stimulation

**Figure 2:**
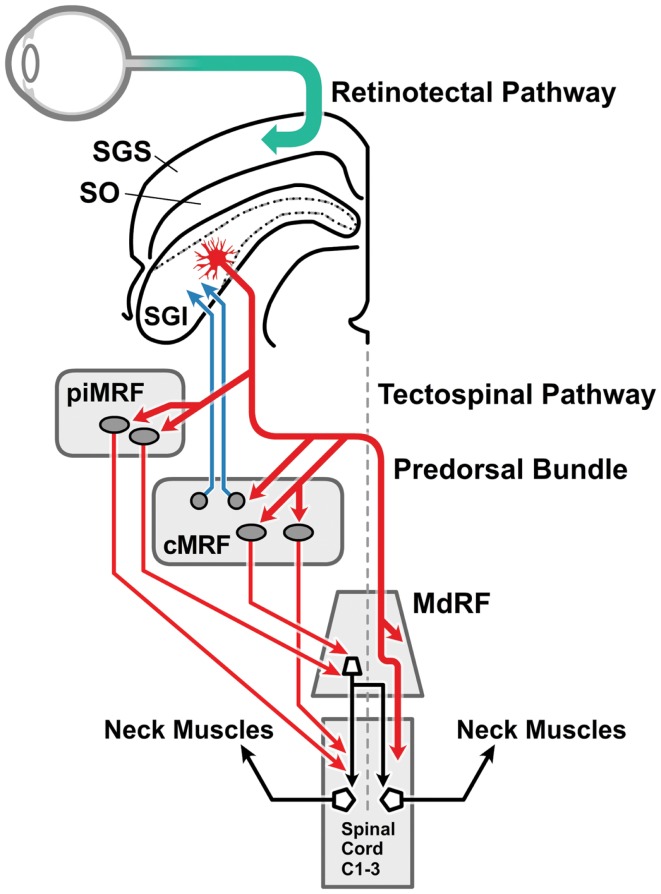
**The visual head-turn pathway: the cephalomotor premotor pathway from the intermediate and deep lamina of the superior colliculus**. This diagram [adapted from Ref. (76) with permission] illustrates the brainstem pathways involved in the head turn in response to a salient environmental change. Magnocellular retinal ganglion cells form the retinotectal pathway (green arrow) to visual sensory cells in the SGS of the superior colliculus. The direct connection between the superficial and the intermediate and deep laminae of the superior colliculus (not drawn) is illustrated in Figure 4. The cephalomotor premotor neurons, originating in the SGI of the superior colliculus, form the tectospinal and tecto-reticulospinal tracts to reach the motor neurons in the upper cervical cord by two routes: (a) direct pathway (thicker red arrow): the decussating predorsal bundle pathway (medial longitudinal fasciculus in man) can access the upper spinal cord directly or via the MdRF; (b) indirect pathway (thinner red arrows): via (i) the rostral portion of the MRF adjacent to the interstitial nucleus of Cajal (piMRF, vertical gaze), and (ii) the caudal portion of the MRF, the central mesencephalic reticular formation (cMRF, horizontal gaze). The cMRF also provides feedback to the superior colliculus (blue arrows) in relation to head and gaze position. MdRF, medullary reticular formation; MRF, mesencephalic reticular formation; cMRF, central mesencephalic reticular formation; piMRF, peri-interstitial nucleus of Cajal mesencephalic reticular formation; SGI, stratum griseum intermediale; SGS, stratum griseum superficiale; SO, stratum opticum.

In macaques, anatomical studies have shown that projections to downstream oculomotor and neck muscle structures originate in separate laminae of the stratum griseum intermediale in the DLSC (63, 64) (Figure 2). *The tectospinal tract* is a very conserved structure present in all mammals, although it is better developed in predatory than in less predatory species (65, 66). It originates in the DLSC and terminates almost exclusively on inter-neurons in the upper cervical cord whose motor neurons innervate neck muscles, and in the lower cervical enlargement innervating the forearm and hand muscles (65, 67, 68). Another important tectospinal target is the propriospinal neurons in the C3–C4 segments of the cervical cord, which govern target reaching of the forelimb (69). *The tecto-reticulospinal tract* also originates in the DLSC of the SC, involves the tegmental and pontine reticular formation and also ends in the upper cervical spinal cord (67, 68, 70–73) contacting, at least in part, the motor neurons of head extensor muscles. The cells of origin of the tectospinal and the tecto-reticulospinal tracts overlap to some degree in the DLSC (73). Electrical stimulation of the DLSC and the dorsal tegmentum in the cat activates reticulospinal neurons (74) that in turn activate motor neurons of the flexor and extensor muscles of the hind and forelimb (75). In the macaque, tecto-reticulospinal pathways to the neck and hand muscles have been demonstrated (76); neck muscle EMG activity following a visual stimulus is conveyed by the tecto-reticulospinal pathways (77). In cats, this pathway displays bursts of activity time-locked to visual target presentation (78). Similar burst discharges in response to visual stimuli have been found in tecto-reticular neurons in rats (79).

## Visual Response in the Superior Colliculus

**Figure 3:**
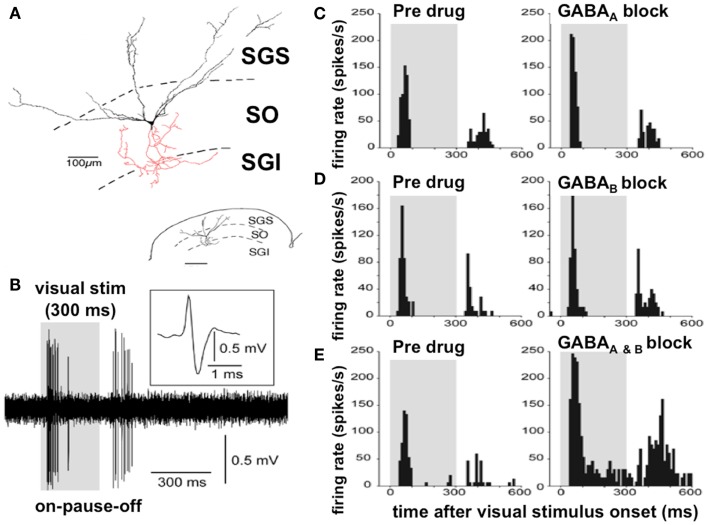
**Discharge properties of the wide field vertical cells in the superficial lamina of the superior colliculus: GABA_A_ and GABA_B_ receptors cooperatively shape transient “ON” responses**. **(A)** A typical WFV cell is illustrated in the SGS and SO; both form part of the superficial laminae of the superior colliculus, contacting a premotor neuron (in red) in the SGI (part of the DLSC). Inset: small-scale drawing of same section. **(B)** Typical ON–PAUSE–OFF pattern response in a WFV cell to a static visual stimulus lasting 300 ms. Five traces are superimposed. The inset shows the expanded trace of a single spike with a positive–negative sequence due to close apposition of the recording electrode to the cell. Gray-shaded areas in the panels indicate duration of visual stimulus presentation. **(C)** Local application of the GABA_A_ receptor antagonist significantly increased the peak-firing rate but did not affect “ON” response duration. **(D)** Local application of the GABA_B_ receptor antagonist significantly prolonged the “ON” response duration, but did not affect the peak-firing rate. **(E)** Local application of both GABA_A_ and GABA_B_ receptor antagonists significantly increased the peak-firing rate and prolonged the “ON” response duration. Figure adapted from an original study kindly provided by Professor Tadashi Isa, from Ref. (80) with permission. GABA, gamma-aminobutyric acid; WFV, wide field vertical; SGS, stratum griseum superficiale; SGI, stratum griseum intermediale; SO, stratum opticum; DLSC, intermediate and deep laminae of the superior colliculus.

Among others, the Okazaki laboratory of Tadashi Isa has been extremely active in examining the microcircuitry of the superior colliculus (Figure 3). The predominant cell type in the superficial layer of the superior colliculus is the wide field vertical (WFV) cell, mainly in the stratum opticum of the SLSC; these cells have characteristic dendritic trees and are luminance and motion sensitive (Figure 3). In response to a visual stimulus, most of the neurons in the SLSC exhibit transient “ON” responses within 50 ms of the stimulus onset (80). With a persistent visual stimulus, most of these cells enter a pause phase and then only discharge again when the visual stimulus is switched off. This sequence “ON–PAUSE–OFF” (Figure 3) in the WFV neurons is characteristic of the response in the superior colliculus to the switching on and off of the visual stimulus or the response to movement. This is the mechanism by which the superior colliculus acts as a detector of salient stimuli (Figure 3).

### GABAergic inhibition in the superior colliculus and normal temporal discrimination

Application of a GABA_B_ antagonist to an electrode recording SLSC neuronal responses to optic fiber inputs in mice prolongs the “ON” burst duration. With blocking of both GABA_A_ and GABA_B_ receptors, there is excessive and prolonged burst activity in both the “ON” and “OFF” phases with loss or attenuation of the normal “PAUSE” phase (80) (Figure 3).

GABAergic SLSC inter-neurons, following a visual stimulus, activate GABA_B_ receptors and limit the duration of both “ON” and “OFF” responses (81, 82). GABAergic inhibition of activity in excitatory neurons prevents unnecessarily prolonged burst activities in the SLSC local circuit, which affects the burst firing in the DLSC through direct signal transmission from the superficial to the deeper layer of the SC (24, 81, 83, 84). This mechanism may endow the SLSC with the ability to detect the appearance, but not the persistent presentation, of an object in the visual field, allowing the SLSC to function as a saliency detector, which might be modulated by cholinergic inputs (85).

### Mechanism of abnormal temporal discrimination in dystonia

Excessive firing in both the “ON” and “OFF” phases of the visual response in the SLSC and the consequent reduction of the “PAUSE” phase, caused by loss of GABA inhibition, results in increased discharge duration and loss of sharpness of the offset of the neuronal response (Figure 3). The effect of the increased duration of both the “ON” and “OFF” phases of the discharge in the WFV cells would increase the duration of the inter-stimulus interval before two asynchronous stimuli would seem, to the observer, as being separate in time. Thus, we postulate that impaired GABAergic activity in the SLSC would result in an inability to discriminate two visual stimuli in short succession and result in abnormal (prolonged) temporal discrimination.

## Control of Cephalomotor Output from the Superior Colliculus

### Disinhibition of the superior colliculus and cephalomotor neurons

**Figure 4:**
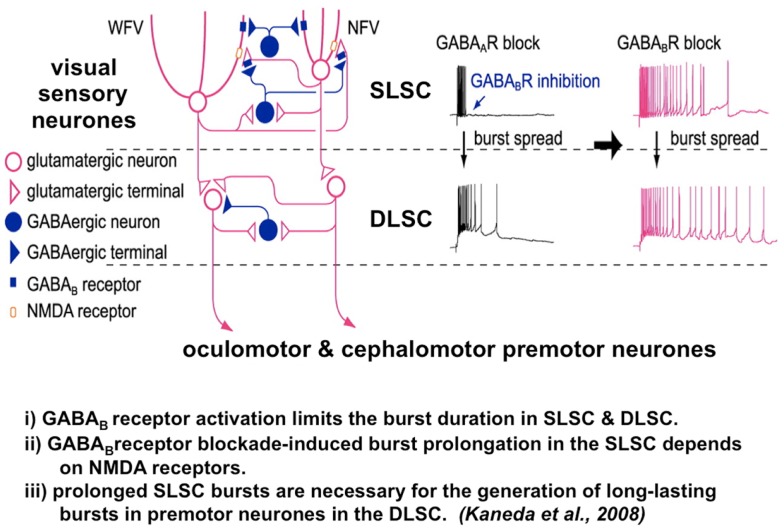
**The microcircuit of the superior colliculus. Reduced GABA_B_ inhibition causes pronged burst duration firing in both the sensory and premotor neurons. A postulated mechanism for both abnormal temporal discrimination and cervical dystonia**. Diagram of the local circuit underlying GABA_B_ receptor-mediated regulation of bursts in the superior colliculus [adapted from an original study kindly provided by Professor Tadashi Isa, from Ref. (82)]. Post-synaptic GABA_B_ receptors expressed in both NFV and WFV cells and presynaptic GABA_B_ receptors located on glutamatergic synaptic terminals in the SLSC are activated by synaptically released GABA during bursts of SLSC GABAergic neurons. GABA_B_ inhibition normally curtails the discharge of both the sensory and premotor neurons. When GABA_B_ receptors are blocked, burst duration in sensory neurons, the SLSC, may be prolonged in an NMDA receptor-dependent manner. Under conditions of reduced GABA_B_ inhibition, this prolonged bursting may spread to the DLSC involving the oculomotor and cephalomotor premotor neurons. This prolonged bursting is postulated to result in excessive cephalomotor neuronal activity and result in cervical dystonia. DLSC, intermediate and deep laminae of the superior colliculus; GABA, gamma-aminobutyric acid; NMDA, *n*-methyl-D-aspartate; NFV, narrow field vertical cell; SLSC, superficial lamina of superior colliculus; WFV, wide field vertical.

The vertical interlaminar connection from the SLSC to the DLSC requires disinhibition from the GABAergic system for the signal to transmit in the pathway (24, 86) (Figure 4). The relationship between GABAergic inhibition from the SNpr and the intrinsic GABAergic inter-neurons of the superior colliculus has been examined by Isa and his colleagues. Neuroanatomical studies have shown that SNpr input to the superior colliculus contacts both glutaminergic motor cells in the DLSC and GABAergic inter-neurons. GABA_B_ receptor activation limits both the burst duration of the visual cells in the SLSC and the premotor neurons in the DLSC. Importantly, prolonged bursts in the visual sensory cells in the SLSC, caused by GABA_B_ receptor blockade, are necessary for the generation of long-lasting bursts in the premotor neurons in the DLSC (Figure 4) (81, 82).

### Disinhibition of the superior colliculus in the generation of experimental cervical dystonia in primates

In monkeys, experimental evidence has shown over the last 25 years that (a) unilateral SNpr lesions induced head turning/tilting to the opposite side (38), (b) muscimol (a GABA agonist) injection into the left SNpr induced a severe contralateral torticollis in 10/12 injections (87), and (c) intranigral muscimol induced a contralaterally directed torticollis from central and posterior sites of the SNpr in 3/3 monkeys (88).

Further to their initial experiments, Karen Gale’s group examined the effect of blocking the DLSC (89). In four macaques pretreating the DLSC with muscimol, before lesioning the SNpr in the same animal, prevented or attenuated the development of the experimentally induced cervical dystonia (laterocollis in three animals and torticollis in one). Thus, reduction of the inhibitory GABAergic input from the SNpr to the superior colliculus in macaques causes a movement disorder resembling cervical dystonia. Blocking the motor output from DLSC by pretreating the superior colliculus prevents the movement disorder (89).

## Disinhibition of the Superior Colliculus Causes Both Dystonia and Abnormal Temporal Discrimination

A core neurophysiological feature of dystonia is reduced inhibition at all levels of the CNS; the most probable cause of this is defective GABAergic inhibition (90). It thus might be reasonably postulated that the link between abnormal temporal discrimination and cervical dystonia is reduced GABAergic inhibition affecting the superior colliculus. Such impaired inhibition results in increased excitability and abnormal burst firing of visual sensory neurons in the SLSC; the subsequent increased excitability of the cephalomotor cells of the DLSC causes experimental cervical dystonia. Thus, it would be reasonable to postulate that abnormal temporal discrimination in both patients with cervical dystonia and their unaffected first-degree relatives is a subclinical marker of defective GABA inhibition in the superior colliculus.

## Cerebellar Lesions, Dystonia, and Temporal Discrimination

The role of cerebellar lesions in the genesis of secondary dystonia including cervical dystonia and blepharospasm has been recently highlighted (91, 92). Temporal discrimination is abnormal in cerebellar degeneration (93). It is likely that inputs from the cerebellum modulate the intrinsic microcircuit of the superior colliculus but the mechanisms have not been examined in animal models.

## Clinical Evidence of Abnormal Head-Turn Control in Cervical Dystonia

In the last few years, a number of investigators have demonstrated abnormalities in head control and head turning in cervical dystonia. Given the difficulties with rapid alternating movements seen in dystonic muscles, this might not be unexpected. However, foveation delays, head on trunk bradykinesia, and truncal bradykinesia, exceeding that found in Parkinson’s disease, are seen in cervical dystonia (94). Oculomotor function is normal in cervical dystonia but gaze control (combined head and eye movement) is defective because abnormal head turning interferes with saccadic function. A number of investigators have implicated the midbrain interstitial nucleus of Cajal in this defective mechanism (95, 96) but the preponderance of the experimental evidence described above indicates that defective inhibition within the superior colliculus and from the SNpr, disrupting the midbrain attentional orienting network, is the more probable mechanism of disease pathogenesis in cervical dystonia.

## Other Late-Onset Focal Dystonia Phenotypes

Most work quoted in this study has addressed cervical dystonia and the cephalomotor system. However, it is likely that all the adult-onset focal dystonia phenotypes share similar etiological factors and pathogenic mechanisms (97). Anatomical studies indicate that premotor neurons originating in the DLSC form part of the tecto-reticulospinal and tectospinal tracts, and may affect facial, bulbar, forearm, and hand muscles, and thus be implicated in the genesis of cranial and focal hand dystonias (69, 76, 98–101) (Figure 2). Certainly abnormal temporal discrimination is found in all these focal dystonia phenotypes suggesting that similar pathogenic mechanisms may be involved (7, 102, 103).

## Conclusion

We postulate that cervical dystonia and abnormal temporal discrimination are both due to a disorder of the midbrain network for attentional orienting, caused by impaired GABAergic mechanisms of inhibition of sensorimotor processing within the superior colliculus. This deficiency in GABAergic activity results in abnormal burst firing in the visual sensory cells in the superficial laminae of the superior colliculus, and thus the abnormal temporal discrimination found in patients with cervical dystonia and their unaffected first-degree relatives. Prolonged duration firing of the visual sensory neurons is a necessary prior abnormality for the development of the hyperexcitability of the premotor neurons in the DLSC. These hyperexcitable DLSC premotor neurons, via the tecto-reticulospinal and tectospinal pathways, stimulate motor neurons in the upper cervical spinal cord resulting in the abnormal, jerky head spasms characteristic of cervical dystonia.

## Problems and Future Solutions

Clearly, this hypothesis does not explain the cause of the defective GABAergic inhibition in the superior colliculus. It is likely that processes upstream in the basal ganglia in relation to dopamine processing and disordered D1/D2 receptor availability are responsible [reviewed in Ref. (104)].

In order to advance this hypothesis, a number of experiments are required. These experiments include: (a) examination of the visual response to salient stimuli in the neck muscles in patients with cervical dystonia and in their unaffected first-degree relatives with abnormal and normal temporal discrimination. (b) Examination of the fMRI response to looming stimuli in the superior colliculus in both patients with cervical dystonia and their unaffected relatives with and without abnormal temporal stimulation. (c) Magnetic resonance spectroscopic examination studies are needed for levels of GABA in the superior colliculus in patients with cervical dystonia and their relatives with and without abnormal temporal discrimination.

## Conflict of Interest Statement

The authors declare that the research was conducted in the absence of any commercial or financial relationships that could be construed as a potential conflict of interest.

## Abbreviations

AOIFD, adult-onset idiopathic isolated focal dystonia; DLSC, intermediate and deep laminae of the superior colliculus; SLSC, superficial laminae of the superior colliculus; SNpr, substantia nigra pars reticulata; TDT, temporal discrimination threshold; WFV, wide field vertical.
